# AODV-EOCW: An Energy-Optimized Combined Weighting AODV Protocol for Mobile Ad Hoc Networks

**DOI:** 10.3390/s23156759

**Published:** 2023-07-28

**Authors:** Yi Jiang, Hui Sun, Muyan Yang

**Affiliations:** 1 Department of Communications Engineering, Harbin University of Science and Technology, Harbin 150080, China; jasonj@hrbust.edu.cn (Y.J.);; 2 The Higher Educational Key Laboratory for Measuring & Control Technology and Instrumentation of Heilongjiang Province, Harbin 150080, China

**Keywords:** mobile ad hoc network, analytic hierarchy process (AHP), entropy weight method (EWM), route link selection, AODV

## Abstract

The Ad Hoc On-demand Distance Vector (AODV) is a routing protocol for mobile ad hoc networks (MANETs) and other wireless ad hoc networks. The vanilla AODV protocol is simple and easy to implement because it only uses the hop count as a routing metric. Single-metric route determination also causes problems, such as network congestion and energy exhaustion, which limit the usage of AODV in resource-limited applications. To solve these problems, the authors propose a new routing protocol that combines the analytic hierarchy process (AHP), the entropy weight method (EWM), and AODV. The proposed protocol uses energy, congestion, and the hop count as metrics and weights these three metrics using AHP and EWM. To address the importance of energy in applications, such as drones, the proposed protocol chooses different comparison matrices for AHP at different node residual energy levels. Finally, the node chooses the best route link according to the score (sum of weighted metrics). It is also suitable for wireless sensor networks because the proposed protocol considers the residual energy of the node. The simulation results show that the improved routing protocol can effectively reduce the average end-to-end delay and energy consumption and prolong the lifetime of the whole network.

## 1. Introduction

The biggest difference between mobile ad hoc networks (MANETs) and other networks is that MANETs have no infrastructure, and the nodes in the network may have limited resources. Due to decentralization, a characteristic of MANETs, each node is required to constantly sense surrounding nodes and maintain routing information to communicate. The limitation of resources directly limits the communication radius and energy. With the growth in the communication time, the energy of the nodes will be exhausted, and the death of nodes will also cause changes in the network topology and the overall performance of the network.

MANET routing protocols can be categorized into table-driven methods, reactive and on-demand routing protocols, and hybrid methods.

DSDV (destination-sequenced distance vector routing) [1] and WRP (wireless routing protocol) [2] are two typical table-driven routing protocols. The characteristic of table-driven routing protocols is that the node maintains the routing table by itself and may send partial update packets to preserve resources when a change in the network topology is identified. However, the table-driven routing protocol does not scale well with an increase in nodes in the network. Additionally, the periodic broadcasting of messages by this type of routing protocol leads to more energy consumption and a rapid decline in the lifetime of the entire network. Reactive and on-demand routing protocols, such as AODV [3,4] and DSR (dynamic source routing) [5], do not periodically broadcast update messages. Thus, they have reduced overheads and consume less energy to find routes. ZRP (zone routing protocol) [6,7] is a typical hybrid method that combines the characteristics of a priori routing and reactive routing and uses an a priori method locally to reduce the propagation range. ZRP eliminates the long-route request delays and flooding broadcast mechanism by recording only local neighborhood nodes in the table-driven discovery method. For nodes that are not in the local zone, on-demand routing protocol features are used in ZRP. However, the routing protocols presented above are not suitable for mobile and ad hoc wireless environments. Experiment results [8] indicate that networks using the AODV routing protocol have longer lifetimes than those using the other two routing protocols, which is why authors have focused on AODV.

The main contributions of this paper are listed below:

1. The study introduces a multimetric mechanism for AODV to solve the problems of a short lifetime and poor link communication caused by node congestion. Furthermore, the study uses the analytic hierarchy process (AHP) [9] in operations research to carry out link selection according to the metrics.
2. As for the problem of the subjectivity of AHP, the study uses the entropy weight method (EWM) [10] to constrain the objectiveness of the routing weight. Finally, the weights of the metrics are constrained by both AHP and EWM, which is why the method uses combined weighting. Additionally, considering that the combined weights may not reflect the importance of the residual energy of the node, the study introduces a method to choose different weights derived from different comparison matrices, according the level of residual energy in the node. That is why the proposed protocol is known as AODV-EOCW (AODV with Energy-Optimized Combined Weights).
3. In this study, simulation experiments were conducted to compare the routing protocols again, and the experiment results indicate that the new routing algorithm can improve the network’s lifetime and reduce the average end-to-end delay.

## 2. Related Work

AODV is a widely accepted reactive and a priori routing protocol. In AODV, when the target node receives the first RREQ (routing quest) routing request message from the source node, the link is considered stable, and the RREP (routing reply) message is returned to establish a complete routing link. Then, if the RREQ message from another link from the same source node is received again within a certain time period, the destination node will discard it. This reduces the delay and message forwarding processing to a certain extent and achieves the goal of saving energy. However, the routing table of the AODV routing protocol only stores one routing path, which means that the link will be heavily used, and the energy of the nodes on the link will be rapidly exhausted.

The early death of nodes will decrease the performance of the whole wireless network and shorten the lifetime of all nodes. In addition, AODV simply chooses the route by a single metric among many other routing metrics that can reflect the stability of links, which limits the ability of AODV to choose more stable routing links.

In terms of improving the lifetime of ad hoc networks, many researchers have introduced the concept of energy and added corresponding fields in their implementations. Sridhar [11] considered energy during routing selection to improve the routing performance. Wentao [12] proposed that the congestion problem is an indicator of the routing performance; the determination of whether to transmit RREQs is based on the relationship between the cache size and the threshold. Ranjan [13] introduced the backup routing mechanism to estimate the energy of the routing node and the time at which the route path breaks. When the route breaks, the standby route can be started on time. Frikha [14] proposed a new routing protocol based on AODV, in which the most important concept is the energy threshold. When the energy level is lower than a certain threshold, the node can reject data packets to preserve its own energy. The results of the policy indicate that the corresponding changes to the forwarding mechanism of the AODV routing protocol can extend the network’s lifetime. Boudhir [15] proposed Dichotomic-AODV from the perspective of controlling the transmission of packets to reduce the energy consumption. Before the establishment of the routing path, it is most likely that the intermediate node will be required to forward the messages to be transmitted. The new method reduces the broadcast by increasing the probability of discovering the destination and using the dichotomy method to reduce the transmission of route discovery control messages during route establishment, thus reducing the energy consumption. De Rango [16] proposed an ant-colony-based algorithm to allow the discovery of the minimum number of MDR paths required to preserve the energy and balance the data traffic through the round-trip time-delay evaluation on the path from the source to the destination. De Rango’s algorithm is able to satisfy multiple metrics for multiobjective optimization, such as the end-to-end delay, load balancing, and energy saving. Pathak [17] proposed a new route improvement method by selecting paths using the temporal load on the intermediate nodes and by distributing the load among the free nodes during the transmission of data. Darabkh [18] used a method similar to Boudhir [15] to reduce the cost of control messages and extend the network’s lifetime. The difference is that the scene of Darabkh’s research is the Internet of Vehicles. Because the vehicles generally have unlimited energy, the method of directional flooding based on geographical coordinates is possible.

In terms of reducing link congestion in ad hoc networks, the most common method is to introduce new routing metrics to measure the degree of congestion. Baboo [19] proposed a hop-by-hop routing congestion-aware self-organized routing protocol, which comprehensively considers routing metrics from multiple perspectives, such as the route queuing delay, data transmission rate, link communication quality, and MAC overhead. This method partly prevents network congestion. However, there are many factors to be considered in the method, so the logic to be implemented is relatively complex, and the cost of the whole network is high.

Senthilkumaran [20] estimated the congestion degree of nodes based on the average queue length of the route receiving area. At the same time, the node sends an alert to the neighboring nodes according to the congestion degree for a period to select the nodes with low congestion as the next hop. In the same year, Barma [21] adopted a similar method to comprehensively calculate the load on the link by introducing the residual energy of the node and the length of the route receiving queue. Barma proposed the storage of the comprehensive weight value of energy and the congestion of different links in the routing table and selected links according to the comprehensive value. Jabbar [22] further deepened the selection method of multiroute metrics and abandoned the shortest hop number to select the optimal path. Jabbar selected links by using multiple routing metrics and comprehensively evaluating links based on the status of network nodes. Huang [23] introduced the concept of the node set. On the basis of calculating the estimated node energy and lifetime, Huang established a node set with stable communication and selected a stable next-hop node for data transmission. This method improves the routing throughput and reduces the routing overhead. Pu [24] introduced two factors, the link congestion and node energy, to solve the problems of rapid energy consumption and node congestion during data transmission. Pu used the ant colony algorithm to select nodes with more energy and less load for the optimal chain. In addition, Pu screened out nodes that did not meet the conditions by judging whether the nodes met the set energy level and energy consumption conditions during routing maintenance, thus extending the lifetime of the entire network. Chen [25] took the number of neighbors as the condition of link determination in the scenario of rapid changes in network topology caused by rapid node movement.

Introducing multiple metrics for route selection is a good method to improve the route selection, but the difficulty associated with the multiple metric method is the assignment of a weight to each metric. Kumbhar [26] regarded the direction of movement as one of the multiroute metrics but simply assigned weights based on the directionality and reversibility of the direction. Those two metrics cannot reflect changes in topology well. Patsariya [27] proposed a scoring table mechanism but still failed to break away from the constraint of constant factor weights.

In recent years, AHP as a multicriteria decision-making solution has been given more focus by WSN and MANET researchers. Pabani [28] introduced a fuzzy AHP-based method under multicriteria decision-making to make an intelligent routing decision based on objectives, and the method is aimed for selecting relay nodes in underwater sensor networks. Tomar [29] proposed an AHP-TOPSIS-based method to select the next sensor node to charge in a wireless rechargeable sensor network. In that problem, the sensor nodes are supplied power by single or multiple mobile chargers, which are capable of transmitting power to nearby sensor nodes wirelessly. Khalid [30] introduced a cluster head selection algorithm for wireless sensor networks using AHP. In terms of identifying malicious nodes in MANETs, Joseph [31] utilized an RPL and AODV-based protocol, and Divya [32] developed a security-aware congestion control for a sensor network to get rid of selfish nodes’ intentional attempts to cause congestion. Divya used AHP to determine malicious nodes by remaining energy, node potential value, node load factor, and traffic burst rate as decision factors.

This study used a different yet efficient method for the weighting problem. The details of the proposed method are provided in Section 3.

## 3. AODV-EOCW Protocol

The proposed protocol (AODV-EOCW) combines subjective and objective weighting methods to identify the optimal route. AODV-EOCW first calculates the subjective weights with AHP and a comparison matrix and then objectively corrects the subjective weights with EWM to obtain a more scientific weight for route measurement. The AHP and EWM methods are detailed in Section 3.2 and Section 3.3, respectively.

### 3.1. Protocol

**Figure 1:**
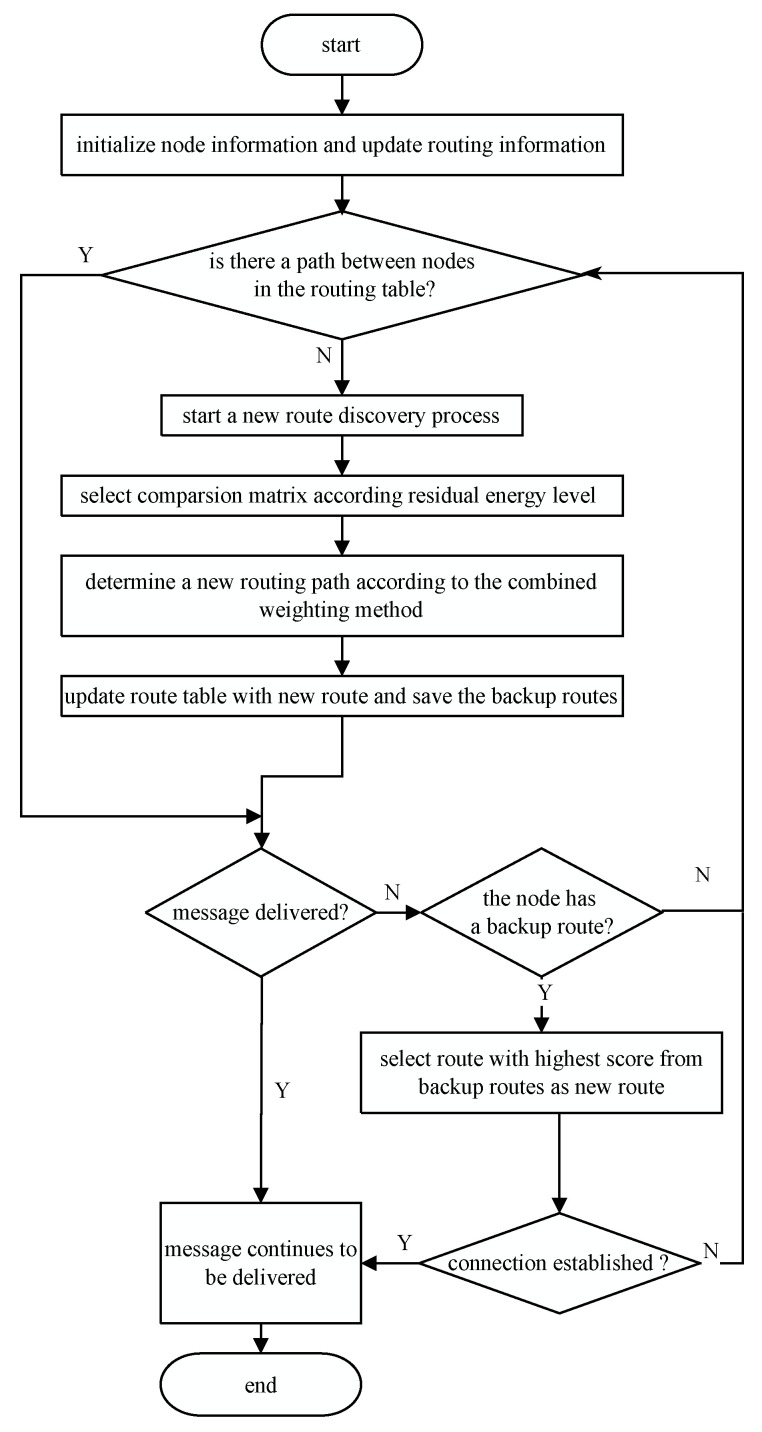
Flowchart of the proposed protocol.

The AODV-EOCW routing protocol is carried out by two stages, namely, the discovery stage and the route establishment stage, as depicted in Figure 1. First, the node initializes itself. Afterwards, if the node is required to send a message, it checks whether there is an entry in the routing table that directly reaches the destination. If there is no routing table entry that directly reaches the destination, the node will perform the route discovery process; otherwise, it will directly send messages according to this routing table entry.

In the route discovery stage, RREQ carries information on parameters such as the congestion, hop count, and energy, so the next hop node can easily obtain the status of the previous hop node.

In the route establishment stage, since the node can identify the status of the previous hop node, the most reasonable path can be calculated using the combined weighting method and the scores of the routing table entries. In the meantime, the node also records the path with a score that is not the highest as the backup route. If the link in the routing table fails, the backup route can be enabled, which can largely reduce the network’s energy consumption and congestion caused by the repeated flooding of RREQ.

### 3.2. Subjective Weighting

The purpose of using AHP in the study was to obtain the weights of the three metrics mentioned above. When dealing with decision-making problems in the process of engineering applications and social production, AHP treats a complex, multiobjective decision-making problem as a system and decomposes the goal into multiple goals or criteria, and then decomposes it into several levels of multiple indicators.

**Figure 2:**
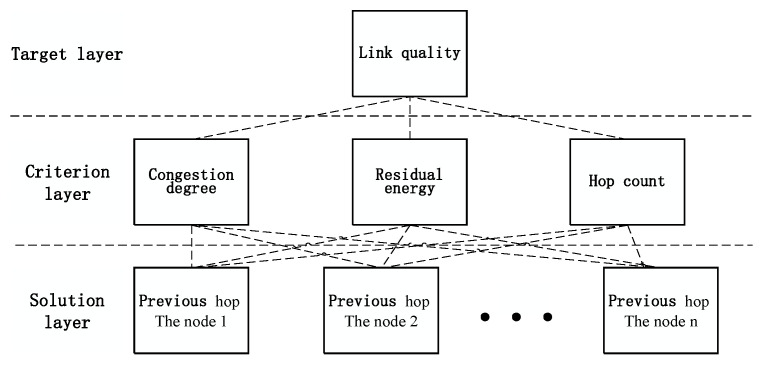
Hierarchy structure of AHP adapted for route selection.

AHP is a hierarchical weight decision-making analysis method based on network system theory and the multiobjective comprehensive evaluation method. This method requires less information, it is simple and practical, and it is widely used in operations research. One disadvantage of AHP is that although it can only be used to select one better solution from existing solutions, it does not provide a new and different solution. However, for routing path selection problems, at any specific time, the node always knows its neighbors, because the last hop node is known, and the route is always selected from the neighbors. This disadvantage has little impact on the routing selection. Before AHP can be adopted for routing path selection, it is first necessary to build a hierarchical structure model and divide it into the target layer, criterion layer, and solution layer, according to their relationships. The hierarchical structure is shown in Figure 2.

The target layer in Figure 2 is used for decision-making and solving problems. In the proposed routing method, the target layer is designed to judge the quality of the communication links between nodes. The criteria layer contains the factors and criteria that need to be considered when judging the target layer, namely, the node congestion, hop count, and residual energy. The solution layer contains the alternatives for decision making, that is, the different communication links between each node and the previous hop. When determining the weights among the factors at different levels, the consistency matrix method [9] is adopted. The purpose of the consistency matrix is to avoid the difficulty associated with comparing all factors together. Through a pairwise comparison, the difficulty caused by the mutual comparison of multiple factors with different properties can be reduced as much as possible. Afterwards, metrics are evaluated according to their importance, and the evaluated values are used to construct a comparison matrix. Finally, the weight value of each element is determined. The form of the judgment matrix *A* is a square matrix, as shown in Equation (1):

(1) $$A=\left(\begin{array}{cccc} 1 & a_{12} & \cdots & a_{1m}\\ \frac{1}{a_{21}} & 1 & \cdots & a_{2m}\\ \vdots & \vdots & \ddots & \vdots \\ \frac{1}{a_{m1}} & \frac{1}{a_{m2}} & \cdots & 1 \end{array}\right)$$

where *m* is a factor in the standard layer. Since the criterion layer only uses three factors for judging the routing metric, the value of *m* is 3, and the elements in the matrix are usually determined subjectively. $a_{ij}$ indicates the importance of the *i*-th element to the *j*-th element. In this study, the authors used the same method as that used by Saaty [33] to construct the judgment matrix. The values and definitions of each element in the matrix are shown in Table 1; the matrix is adapted from Satty [33] too.

After the consistency matrix has been constructed, the eigenvector and the corresponding eigenvalue $\lambda_{max}$ (the largest eigenvalue of the matrix after normalization) can be obtained. Each element in this eigenvector is a weight of the parameters in each criterion layer.

In addition, in order to prevent the degree of inconsistency caused by subjective experiences from exceeding the allowable range, the algorithm also uses the random consistency ratio CR to check the consistency to ensure that the degree of inconsistency is within the allowable range. Its calculation method is shown in Equation (2):

(2) $$CR=\frac{CI}{RI}=\frac{\lambda_{max}-n}{\lambda_{max}^{\prime }-n}$$

where CI stands for the consistency index, and RI stands for the random consistency index, $\lambda_{max}$ stands for the largest eigenvalue of *A*, and $\lambda_{max}^{\prime }$ stands for the largest eigenvalue of the positive reciprocal matrix of *A*. When CR<0.1, the comparison matrix is considered to have a satisfactory level of consistency; otherwise, fine-tuning is required. The fine-tuned matrix of *A* is $A^{*}$; obviously, $A^{*}$ is a comparison matrix too.

If the largest eigenvalue of $A^{*}$ is *x*, the corresponding eigenvector is w. After normalizing the eigenvector w, the weight vector $z=[w_{1},w_{2},w_{3}]$ can be obtained, where the values represent the weights corresponding to the congestion degree, residual energy, and hop count, respectively.

The comparison matrix *A* (or $A^{*}$) reflects the importance of the metrics. The rows and columns represent the congestion degree, the residual energy, and the number of hops. According to Table 1, $a_{12}=3$ means that the congestion degree is slightly more important than the hop count. This study empirically designed the following comparison matrix to reflect the importance of the residual energy. If the residual energy level is above 80%, the comparison matrix $A_{1}$ is chosen to calculate the weights; if the residual energy level is above 50%, the comparison matrix $A_{2}$ is chosen to calculate the weights; and if the residual energy level is below 30%, the comparison matrix $A_{3}$ is chosen to calculate the weights. The three matrices are shown in Equation (3). Finally, the subjective weights from those three matrices are calculated as ω=[0.5396,0.297,0.1634], ω=[0.637,0.2583,0.1047], and ω=[0.7514,0.1782,0.0704], respectively.

(3) A1=123121213121,A2=135131315131,A3=159151519151

### 3.3. Objective Weighting

This is different from AHP, which relies on subjective experience. EWM is an objective weighting method. Entropy is used in information theory to measure the degree of disorder in a system and is an abstraction of the information uncertainty. The entropy weight method determines the weight by calculating the entropy value. The smaller the uncertainty of the measurement is, the less information is reflected, and the lower the entropy weight is in the evaluation system.

Since the entropy weight is completely composed of objective information, it has strong objectivity. In engineering, the entropy weight value is generally also called a correction coefficient, and the objectivity of the weights of metrics can be improved by modifying the weights according to their entropy weights.

The general entropy weight solution process is as follows:

1. Build an evaluation matrix. The matrix is constructed according to different schemes and index numbers *n*, where $X=(X_{ij})$, in which i=1, 2,⋯m, and j=1, 2,⋯, n. In the study, *m* is the number of different links, and *n* is the number of metrics that the route is calculated on, which is n=3.
2. Data normalization. The data are preprocessed according to the method shown in Equation (4). The purpose of this step is to eliminate the incommensurability between indicators:
  (4) $$Y_{ij}=\frac{x_{ij}-min\left(x_{j}\right)}{max\left(x_{j}\right)-min\left(x_{j}\right)}$$
  in which $Y_{ij}$ is the positive value of the *i*-th scheme on the *j*-th evaluation index.
3. Calculate the index entropy value. The entropy value of the *j*-th index is shown in Equation (5).
  (5) $$H_{j}=\sum_{i=1}^{m}Y_{ij}ln\frac{Y_{ij}}{ln\;m}$$
4. Calculate the index deviation. According to the value of each measurement index, that is, $H_{j}$, the degree of deviation $d_{j}$ of the index *j* can be calculated according to Equation (6).
  (6) $$d_{j}=1-H_{j}$$
5. Calculate the entropy weight. According to the number of index deviations, the entropy weight $\mu_{j}$ of the *j*-th index can be calculated according to Equation (7):
  (7) $$\mu_{j}=\frac{d_{j}}{\sum_{j=1}^{m}d_{j}}$$
  where $0\leq \mu_{j}\leq 1$, $\sum_{j=1}^{m}\mu_{j}=1$.

### 3.4. Route Measurement and Scoring

The choice of routing metric should follow the following criteria:

1. Routing metrics are independent of each other. There is no direct dependency between routing metrics to ensure that the information contained in the routing metrics is not redundant.
2. The selected routing metrics should accurately reflect the basic state of the routing nodes and links. Values obtained in this way can more accurately reflect the real situation of the network.
3. The number of routing metrics should be reasonable and appropriate. Although having a larger number of routing metrics can allow better judgment of the network situation, having more routing metrics will directly lead to an exponential increase in the computational cost.

Based on the above three routing selection criteria, this study used energy, congestion, and routing hops as the routing metrics, and the scoring criteria were selected in accordance with the characteristics of AHP.

#### 3.4.1. Congestion Degree

The “white list” mechanism of the AODV routing protocol often leads to a situation where, once the link has been determined, it cannot easily be changed. This situation will cause the energy of the nodes on the link to be exhausted rapidly. In addition, due to the weakness of the node performance, the receiving and sending queues of the route will be full, resulting in a high packet loss rate and larger delay. Therefore, the study used the congestion degree as one of the routing metrics. The calculation of the congestion degree (CD) is shown in Equation (8):

(8) $$CD=\frac{L_{free}}{L_{all}},\;CD\in \left[0,1\right]$$

where $L_{free}$ is the size of the idle sending queue at a certain moment, and $L_{all}$ is the size of the total sending queue. Therefore, the calculated score must be between 0 and 1, where the higher the score is, the lower the congestion degree of the node is and vice versa.

#### 3.4.2. Residual Energy

The residual energy of a node not only directly reflects the survival of the node, but also affects the lifetime of the entire network. Therefore, energy was selected as the second routing metric factor. The residual energy scoring (RE) is shown in Equation (9):

(9) $$RE=\frac{E_{residual}}{E_{initial}},\;RE\in \left[0,1\right]$$

where $E_{residual}$ is the residual energy of the node at a certain moment, which is a variable. $E_{initial}$ is the total energy of the node at the beginning, which is a constant. Therefore, RE must be between 0 and 1, as CD is. Clearly, the more energy there is left, the higher the score is.

#### 3.4.3. Hop Count

The reason why the number of hops was selected as a routing metric is that if the number of hops is too large, the overhead of the entire network will increase, as will the delay. After fine-tuning based on daily experiences and experiments, this study employed the scoring method, as shown in Table 2, for the hop count.

The scores (RH) presented in Table 2 are in the range of 0 to 1. RH scores in this range are used to ensure that the hop count metric is in the same range as the congestion degree and residual energy to facilitate subsequent calculations. Clearly, the higher the routing hop score, the fewer routing hops required.

### 3.5. Link Selection Based on Combined Weighting

Regarding the algorithm that selects routing from multirouting metrics, the quality of the routing performance depends heavily on the scientificity of the weighting of routing metrics. No matter whether the subjective weighting method represented by AHP or the objective weighting method represented by EWM is used, there are incomplete data in ad hoc environments. A comparison of the single-weighting methods is shown in Table 3.

The combined weighting method overcomes the disadvantages of subjective and objective weighting and includes the advantages of both. Thus, the authors of the current study propose that the combined method can obtain more reasonable weights.

Finally, the multiplicative synthesis method was adopted to solve the problem of whether to assign weights using subjective weighting or objective weighting. The method is described in Equation (10):

(10) $$weight_{i}=\frac{\omega_{i}\mu_{i}}{\sum_{i=1}^{n}\omega_{i}\mu_{i}}$$

where $weight_{i}$ is the combined weight of the calculated indicators, *n* is the number of metrics (n=3), $\omega_{i}$ is the weight calculated by AHP (Section 3.2), and $\mu_{i}$ is the weight calculated by EWM (Section 3.3).

## 4. Routing Table Design

The routing protocol proposed in this study chooses the route according to three metrics, so the routing table and control packets must be modified accordingly. The modified routing table is shown in Table 4.

Since the hop count is already in the AODV routing table, it is only necessary to add the congestion degree and the residual energy of the node to the routing table. Additionally, the authors considered that there is only one routing path for vanilla AODV routing. Once the path fails, the process of route discovery will be repeated, which will increase the routing overhead and energy consumption. The improved routing protocol will also store other paths in the backup route and quickly start the restoration of the route after the failure of the main route.

The RREQ route discovery packet of the improved AODV protocol has three routing factors. Nodes can discover RREQ packets through routing, tell other nodes about their three routing metrics, and calculate the score of the entire path based on this to select the best link. The modified RREQ routing request packet is shown in Table 5.

Similar to the RREQ packet, the RREP packet also needs to be modified. It is also necessary to add the congestion degree and node residual energy to RREP. The packet format of RREP is shown in Table 6.

## 5. Experiments

A list of comprehensive experiments was conducted to evaluate the performance of the AODV-EOCW routing protocol. The experimental results were compared with the vanilla AODV routing protocol and the AODV-UU [34,35] routing protocol from the perspectives of the node movement speed, time variation, and node density. Finally, the authors also compared the end-to-end delay, packet delivery rate, and node survival rate.

### 5.1. Experimental Settings

The parameters of the experiments are listed in Table 7.

The vanilla AODV routing protocol does not take the energy problem into account, so the authors used Equation (11) to calculate the survival rate of nodes for AODV:

(11) $$p=\frac{Num_{live}}{Num_{all}}$$

where $Num_{live}$ is the number of nodes that are alive at the end of the simulation, and $Num_{all}$ is the total number of nodes in the simulation.

### 5.2. Experimental Results and Analysis

In this section, three series of experiments were conducted to show the performance of the proposed protocol. The experiment results were the average of ten runs with different random seeds, and the results were plotted with a confidence interval of 0.95 along with the median value.

#### 5.2.1. Simulation with Different Motion Speeds

**Figure 3:**
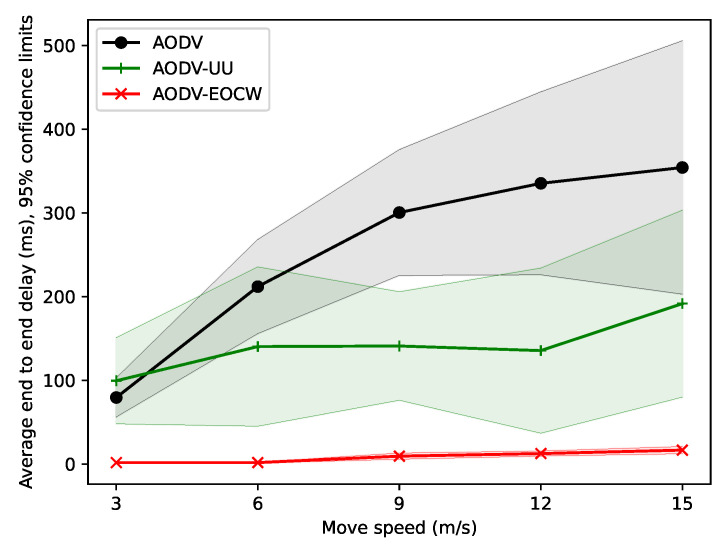
Comparison of the average end-to-end delay.

The number of nodes and the running time were set to 30 and 200 s, respectively, and the speed of the nodes increased from 3 to 15 m/s. The performance of the three protocols was observed. The end-to-end delay between nodes is shown in Figure 3.

It can be seen from the figure that as the nodes move faster, the end-to-end delay is higher in AODV and AODV-UU. Obviously, the two protocols are too sensitive to the movement of the node. The average end-to-end delay of the AODV-EOCW routing protocol also increases significantly, but it still has obvious advantages compared with the other two protocols. The authors believe that this is because the AODV and AODV-UU protocols choose the communication link with the minimum number of hops; as the nodes move faster, the broken links cause AODV and AODV-UU to rebroadcast packets to find new routes.

**Figure 4:**
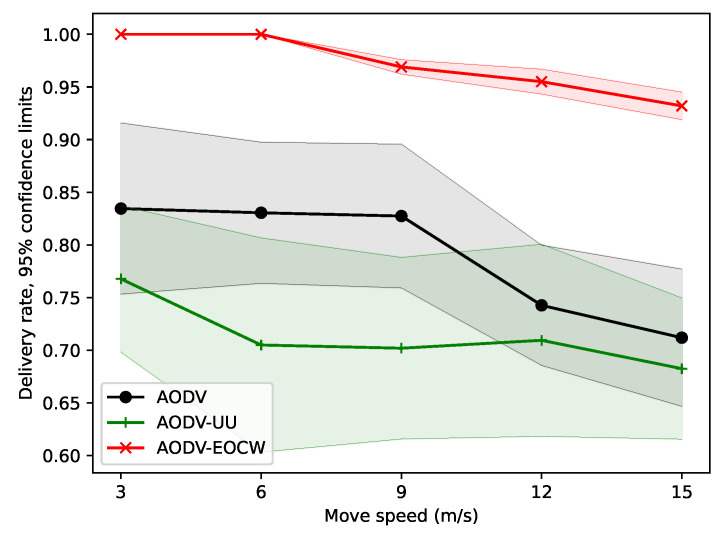
Comparison of the average message delivery rates.

In terms of the message delivery rate, the results are shown in Figure 4. It can be seen from the figure that although the message delivery rates of all three routing protocols show a downward trend as the nodes move faster and faster, AODV-EOCW still exhibits a far better performance than the other two protocols. The main reason for the low message delivery rate of the other two routing protocols is that only routing hops are used as routing metrics. Therefore, it is very easy to cause some routing nodes to be congested and the packet to be lost. Clearly, the better delivery rates of AODV-EOCW are due to the multimetric routing protocol design.

**Figure 5:**
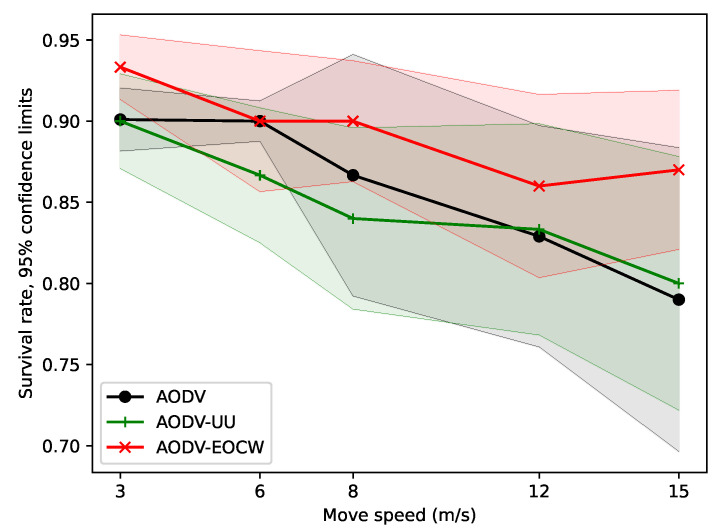
Comparison of average node survival rates.

Finally, survival rate experiments of the same experiment setup are shown in Figure 5. It can be seen that as the nodes move faster, the survival rate of the nodes decreases. The rapid movement of nodes leads to a rapid change in the network topology. The network is prone to link breakages in this situation. Thus, the node sends more control packets to sense the network’s status and also consumes more energy. The experimental results show that the AODV-EOCW routing protocol has obvious advantages.

#### 5.2.2. Simulation with Different Simulation Times

In this section, the simulation time was set from 40 to 200 s to test the long time stability of the protocols. In all experiments, the number of nodes was fixed to 30, and the node movement speed was fixed at 5 m/s.

**Figure 6:**
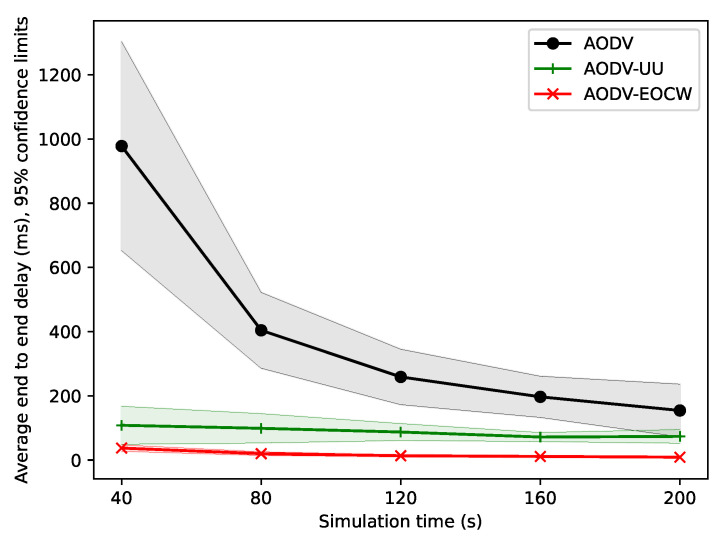
Comparison of the average end-to-end delay.

First, the average end-to-end delay of the three routing protocols is shown in Figure 6. It can be seen from the figure that the average end-to-end delay of the three routing protocols gradually decreased as the simulation time increased, and finally tended to become stable. This is because, at the initial stage, the routing table of each node was empty, the delay of the initial few messages was relatively large, and the average delay was high at the beginning of the simulation. Following the establishment of routes between nodes, the delay of new messages decreased, and the average delay of the network continued to decrease, finally achieving a stable state.

**Figure 7:**
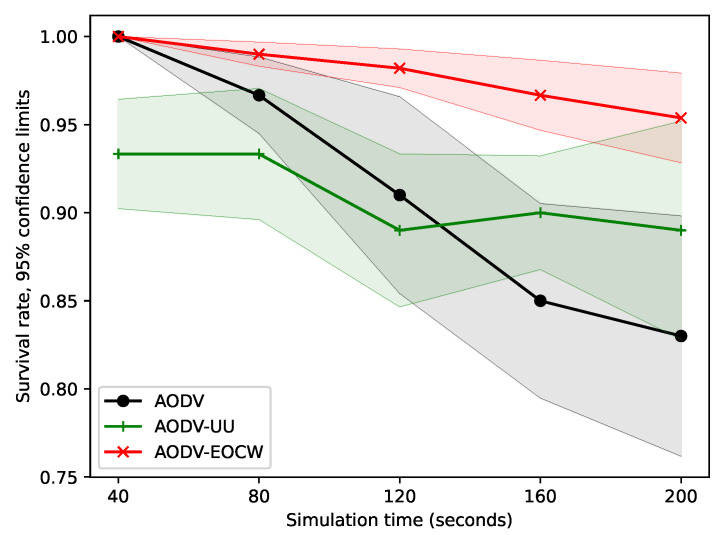
Comparison of the average node survival rates.

The survival rates are shown in Figure 7. It can be seen from Figure 7 that the overall survival rate decreased as the simulation time increased. However, as the network gradually converged, the downward trend of the node survival rate gradually slowed down. Undoubtedly, AODV-EOCW showed a better survival rate again. The reason is that the new protocol takes congestion degree, residual energy, and hop count into account when selecting the next hop, and the relative importance of residual energy among the three metrics will increase as the energy is exhausted, which can be explained by Equation (3).

**Figure 8:**
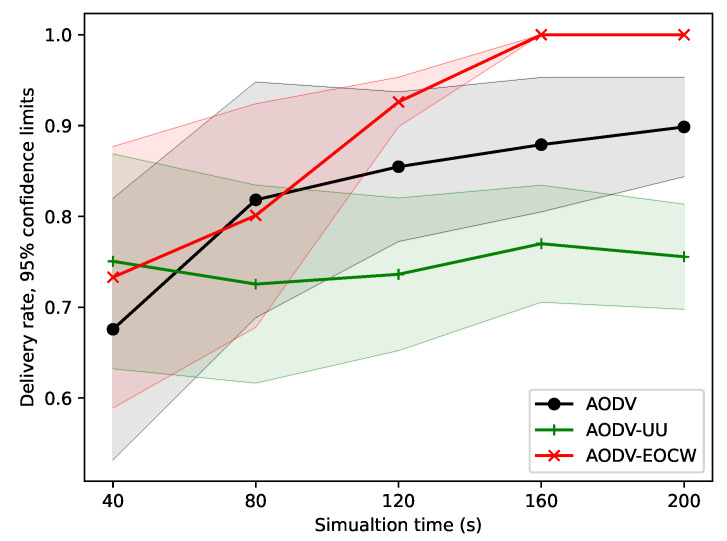
Comparison of the average delivery rates.

Figure 8 shows the variation in the message delivery rate with different simulation times. It can be seen from the figure that as the simulation time increased, the message delivery rate also increased continuously. As shown in the figure, the AODV-EOCW routing protocol outperformed the other two by a large margin. The authors thought that the delivery rates are directly bound up with the nodes’ survival rate. If there are more nodes alive, the more messages can be delivered, so Figure 7 explains the results in Figure 8 from another perspective.

#### 5.2.3. Simulation with Different Node Densities

The experiments presented in this section were designed to test how the node density influences the performance of the routing protocol. In all experiments presented in this section, the nodes were moving at a speed of 5 m/s, and the simulation time was set to 200 s. The number of nodes increased from 20 to 40 at intervals of 5.

**Figure 9:**
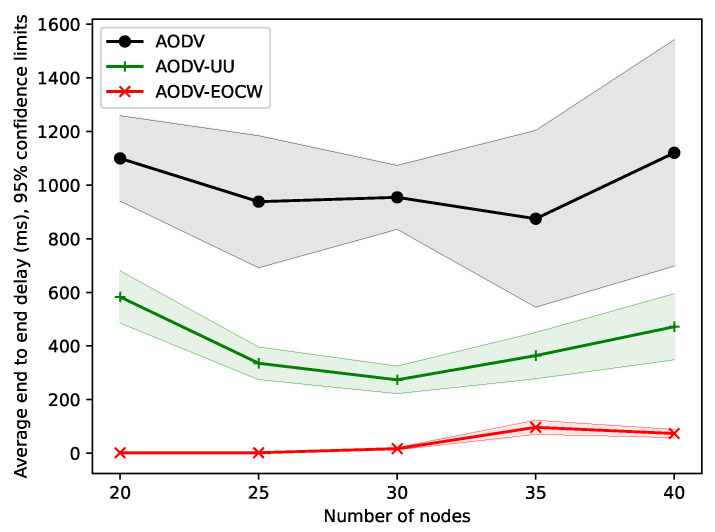
Comparison of the average end-to-end delay.

Figure 9 shows the average end-to-end delay for different numbers of nodes. It can be seen that the trend for the average end-to-end delay of the three routing protocols was a decrease at the beginning and then an increase soon afterwards. The reason for this is that when there are fewer nodes, as the number of nodes increases, the number of candidate links will increase too; thus, the message can be easily delivered.

As the number of nodes further increases, nodes broadcast more and more control messages, which eventually gives rise to congestion. This also occurs when there are too many nodes. In addition, intermediate nodes will also forward messages from other nodes to the next node, which will further intensify the congestion and delay. It can also be concluded that the AODV protocol is suitable for scenarios in which the topology changes rapidly and there are fewer nodes. The advantages of the new routing protocol are more obvious when the number of nodes is small.

**Figure 10:**
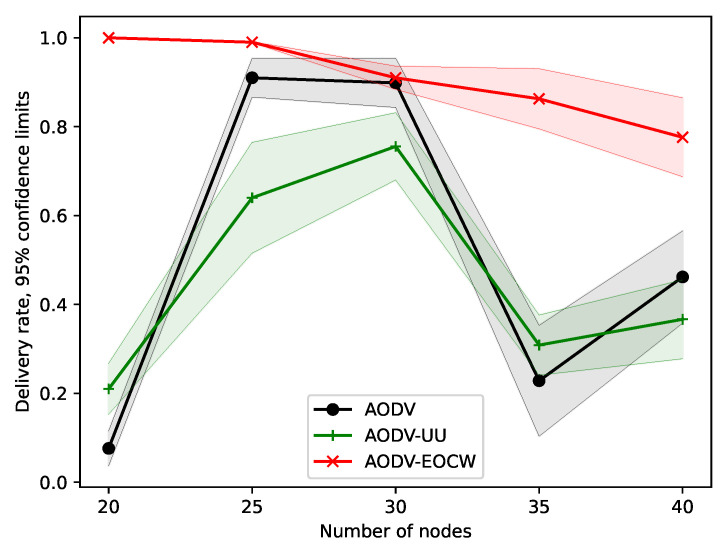
Comparison of delivery rates.

The message delivery rates are plotted in Figure 10. The curves of the three protocols have similar shapes. The difference is that AODV-EOCW exhibits a far better performance in terms of the delivery rate as the number of nodes increases. The authors believe that this is because AODV-EOCW chooses the route according to not only the hop count but also the congestion degree. When there are more nodes in the radio range, AODV-EOCW will choose the noncongested node as the next hop.

**Figure 11:**
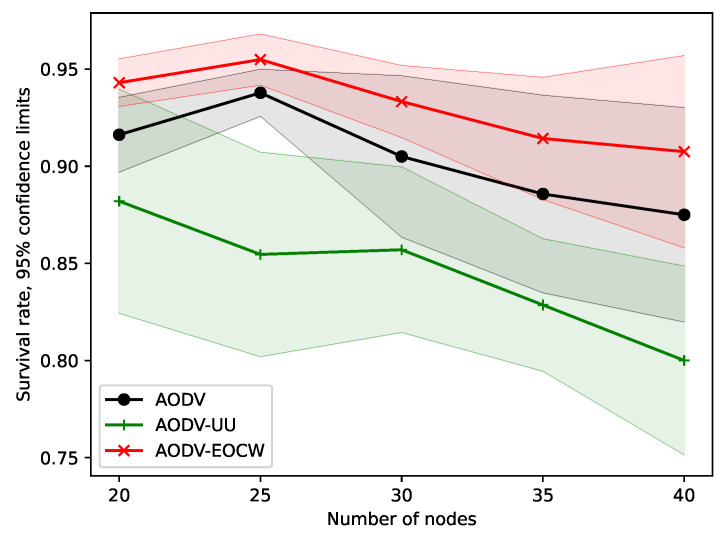
Comparison of survival rates.

The survival rates are plotted in Figure 11. It can be seen from the figure that the survival rates of the three protocols declined rapidly as the number of nodes increased. AODV-EOCW was shown to have a similar survival rate with AODV, and this was far better than that of AODV-UU. The experimental results show that AODV-based protocols consume energy faster in scenarios with a higher node density, and AODV-EOCW has significantly improved the energy efficiency by dynamically changing the judgment matrix according the residual energy.

#### 5.2.4. Simulation of Node Residual Energy and Routing Overhead

In this section, the average residual energies of the nodes are compared between AODV, AODV-UU, and AODV-EOCW protocols. The experimental settings are the same as in Table 7 with the exception that the nodes’ initial energy is set to 5 J to make all nodes alive during the simulation. The simulation time was set to 100 s, all nodes were moved with a speed of 5 m/s, and the number of nodes was 40.

**Figure 12:**
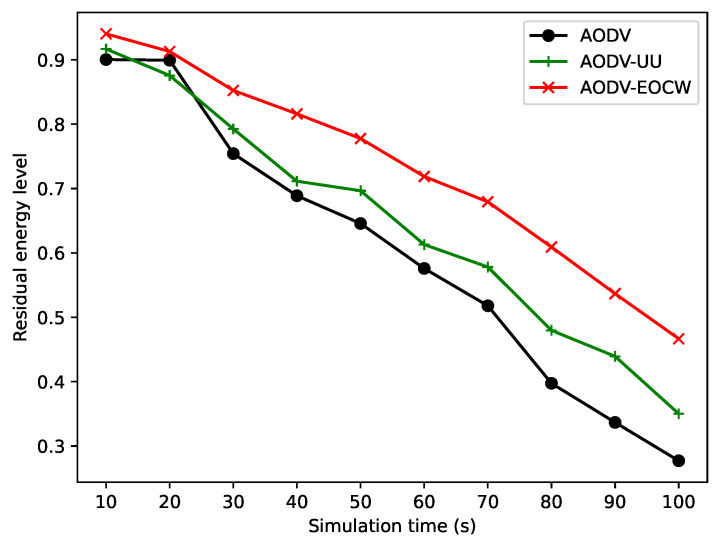
Comparison of residual energy.

Figure 12 illustrates the residual energy level between protocols. As we can see from the figure, the average residual energy levels decrease as the simulation goes on. In the simulation, AODV-EOCW outperforms AODV and AODV-UU unexpectedly. This is because AODV-EOCW can select a route path effectively and thus broadcast fewer packets for route discovery.

**Figure 13:**
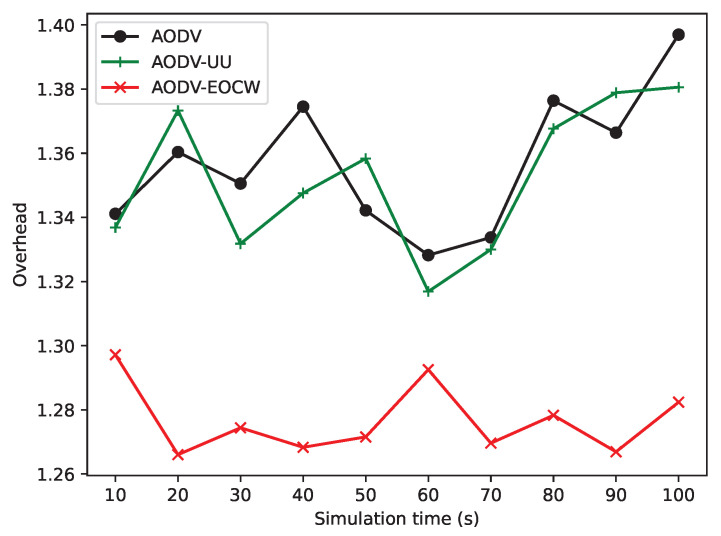
Comparison of routing overhead.

Figure 13 shows that AODV-EOCW outperforms the other two protocols in the routing overhead. As Figure 13 shows, the routing overhead of AODV-EOCW is slightly better than the other, but the three protocols remain in the same level, which is because AODV-EOCW is based on the AODV protocol; although the RREQ and RREP packets are modified to carry more information, the AODV-EOCW protocol broadcasts fewer packets than the other two for route discovery because it selects a better route than AODV and AODV-UU.

## 6. Conclusions

Experiments on the average end-to-end delay, packet delivery rate, and node survival rate were conducted to verify the performance of AODV-EOCW against those of vanilla AODV and AODV-UU. The simulation results show that AODV-EOCW has obvious advantages over the two routing protocols, and it can effectively improve the survival rate and lifetime of the network by selecting a less congested and more stable link to forward packets. According to the experiment results, the AODV-EOCW protocol is suitable for both MANET and WSN applications. Finally, there are two major limitations in this study that could be addressed in future research. First, the residual energy level thresholds that were used to select judgment matrices are designed empirically. How to select the thresholds at runtime will be our focal research point. Second, the proposed protocol has not been verified in real applications. The authors are considering implementing the AODV-EOCW protocol in a homemade mobile sensor node network to investigate how the protocol performs in real applications.

## Figures and Tables

**Table 1:** Explanation of $a_{ij}$ in Equation (1).

$a_{i,j}$	Meaning
1	*i* and *j* are equally important
3	*i* is slightly more important than *j*
5	*i* is more important than *j*
7	*i* is more important than *j*
9	*i* is much more important than *j*

**Table 2:** Routing hops rating.

Routing Hops	Score
1–2	1
3–4	0.6
5–6	0.4
≥7	0.1

**Table 3:** Comparison of different weighting methods.

Weighting Method	Pros	Cons
Subjective weighting method (i.e., AHP)	Determines the weight based on experience, which can better reflect the actual situation	Weight determination has nothing to do with data and relies too much on experience
Objective weighting method (i.e., EWM)	The weight is determined by data and an algorithm and effectively reflects the change in data	Ignores the importance of experience, so data changes will cause greater weight changes

**Table 4:** Routing table.

Routing Table Entries
Destination node’s IP address
Valid flag of the destination node’s serial number
Network interface
lifetime
Destination node’s serial number
Next hop node
Precursor list
Hop count
Backup route
Routing flag
Congestion degree
Node residual energy

**Table 5:** RREQ packet.

RREQ Packet Fields
RREQ ID
Destination node’s IP address
Destination node’s serial number
Source node’s IP address
Source node’s serial number
Congestion degree
Node residual energy
Hop count

**Table 6:** RREP packet.

RREP Packet Fields
Destination node’s IP address
Destination node’s serial number
Source node’s IP address
Source node’s serial number
Congestion degree
Node residual energy
Hop count

**Table 7:** Experiment parameters.

Parameter	Value
Wireless module	Ieee80211ScalarRadioMedium
Motion module	MassMobility
Energy module	StateBasedEpEnergyComsumer
Visualization module	IntegratedCanvasVisualizer
Node’s initial energy	1 J ∼5 J
Shutdown threshold	0.1 J
Antenna’s transmission power consumption	3 mW
Node’s range of motion	1000 m × 1000 m
Receive buffer size	17
Limit of retransmission times	7
Direction of movement	Random

## Data Availability

Not applicable.
